# Supplementary figures and images for: Multiscale model of integrin adhesion assembly
Source: PLoS Comput Biol. 2019 Jun 4;15(6):e1007077. doi: 10.1371/journal.pcbi.1007077 (PMC6568411; doi:10.1371/journal.pcbi.1007077)

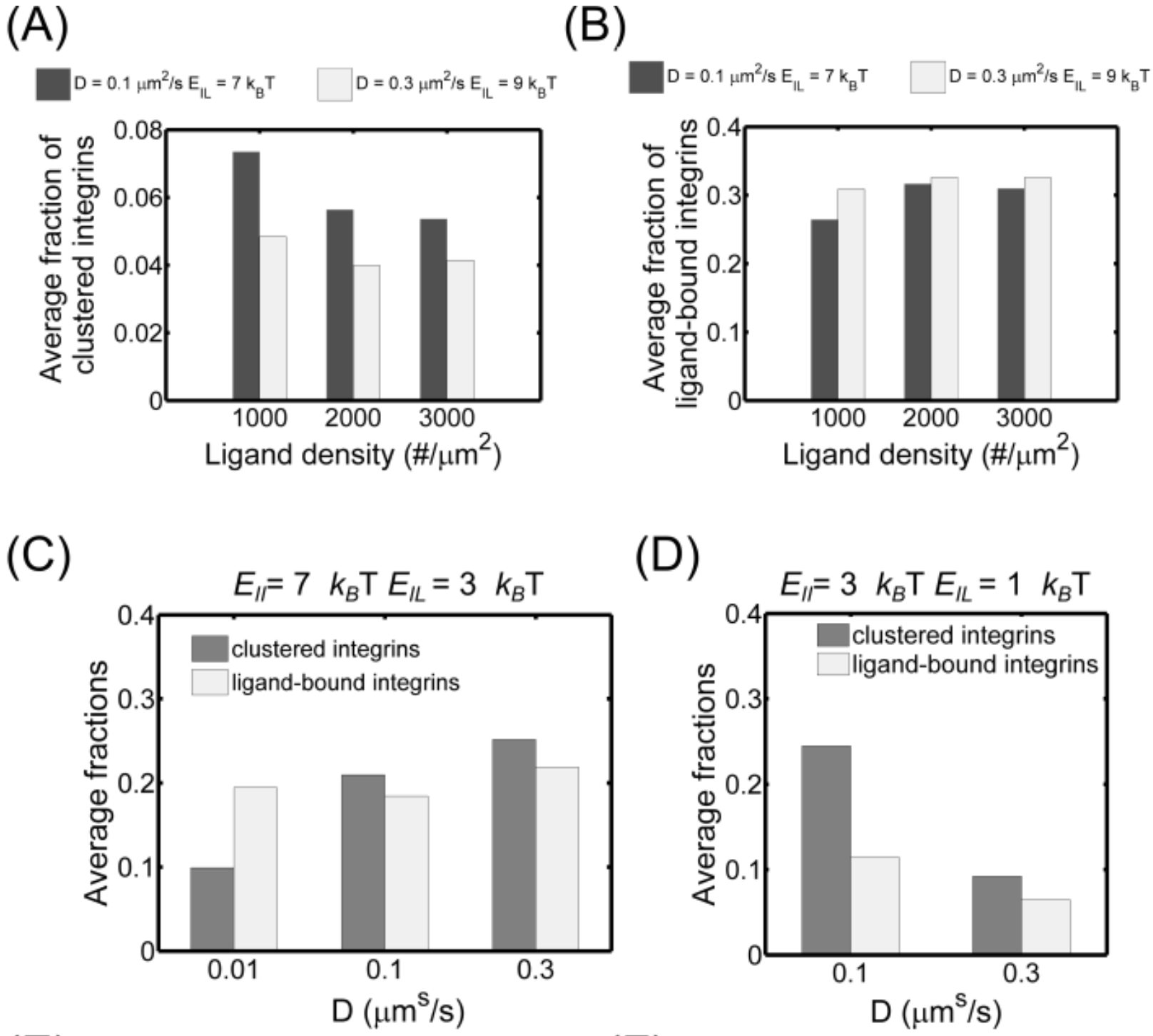

Supplement: S1 Fig — (A) Average fraction of clustered integrins and (B) corresponding fraction of ligand-bound integrins varying ligand density for two conditions of integrin properties: D = 0.1 μm2/s, EIL = 7 kBT, EII = 9 kBT; D = 0.3 μm2/s, EIL = 9 kBT, EII = 9 kBT. Data are computed between 80–100 s of simulations from three independent runs. (C) Average fractions of clustered and ligand-bound integrins varying diffusion coefficient, using EIL = 3 kBT and EII = 7 kBT. Data are computed between 200–600 s of simulations, from three independent runs. (D) Average fractions of clustered and ligand-bound integrins at D = 0.1 and 0.3 μm2/s, EIL = 1 kBT and EII = 3 kBT. Data are computed between 200–600 s of simulations, from three independent runs. (TIF) [file pcbi.1007077.s001.tif]

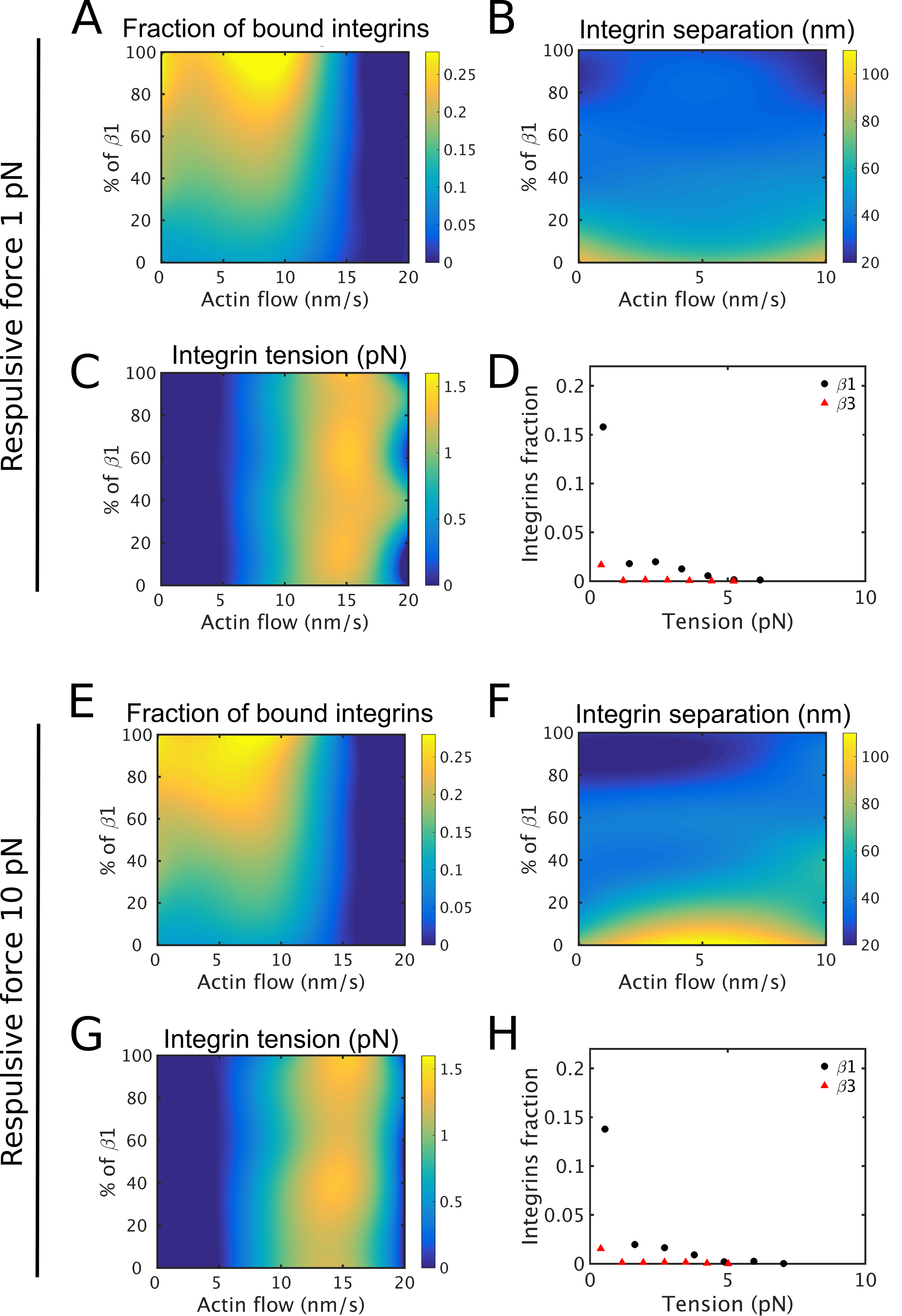

Supplement: S2 Fig — (A) Average fraction of ligand-bound integrins as a function of actin flow speed and percentage of β-1 integrins in a system of β-3 integrins. (B) Corresponding average nearest neighbor distance between ligand-bound integrins. (C) Corresponding average tension per integrin. (D) Distribution of tension on ligand-bound integrins for the two integrins types, using 80% β-1 and 20% β-3 integrins and 10 nm/s actin flow. Data are computed between 1–20 s of simulations, using a weak repulsive potential (1 pN) between integrins closer than 1 nm. Panels E-H show data as in panels A-D for implemented repulsive forces of 10 pN. (TIF) [file pcbi.1007077.s002.tif]
